# P-2029. Results from a Phase 1 First in Human Study of Pemivibart: An Extended Half-Life Monoclonal Antibody (mAb)

**DOI:** 10.1093/ofid/ofae631.2185

**Published:** 2025-01-29

**Authors:** Anna Holmes, Yong Li, Deepali Gupta, Ed Campanaro, Lida Mehr, Anuja Raut, Amanda Copans, Kristin Narayan, Kathryn Mahoney

**Affiliations:** Invivyd, Inc., Waltham, MA; Invivyd, Inc., Waltham, MA; Invivyd, Inc., Waltham, MA; Invivyd, Inc., Waltham, MA; Lida Mehr Consulting, LLC, MIssion Viejo, California; Certera, Inc., Radnor, Pennsylvania; Invivyd, Inc., Waltham, MA; Invivyd, Inc., Waltham, MA; Invivyd, Inc., Waltham, MA

## Abstract

**Background:**

Pemivibart (PEM) is a half-life extended recombinant human monoclonal IgG1 antibody that targets the SARS-CoV-2 spike protein receptor binding domain. PEM has been granted emergency use authorization (EUA) for the pre-exposure prophylaxis of COVID-19 in certain adults and adolescents with moderate-to-severe immune compromise.

**Table 1.**
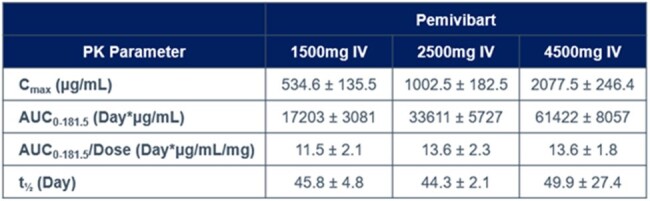
Preliminary Pharmacokinetic Parameter Estimates of Pemivibart [Mean ± SD]

**Methods:**

This is an ongoing Phase 1, first in human, randomized, blinded, placebo (PBO) controlled, single ascending dose study conducted in healthy participants (ppts) aged 18-65 years to evaluate PEM administered by slow IV push. Ppts were randomized 8:2 in one of 3 cohorts (n=8 PEM, n=2 placebo): PEM 1500 mg, 2500 mg, and 4500 mg. The primary objective was to assess the safety and tolerability of PEM. Secondary endpoints included serum pharmacokinetic (PK) parameters (estimated using non-compartmental analysis methodology) and immunogenicity. The trial will continue through 12 months; here we summarize the data through the 6-month timepoint.

**Figure 1:**
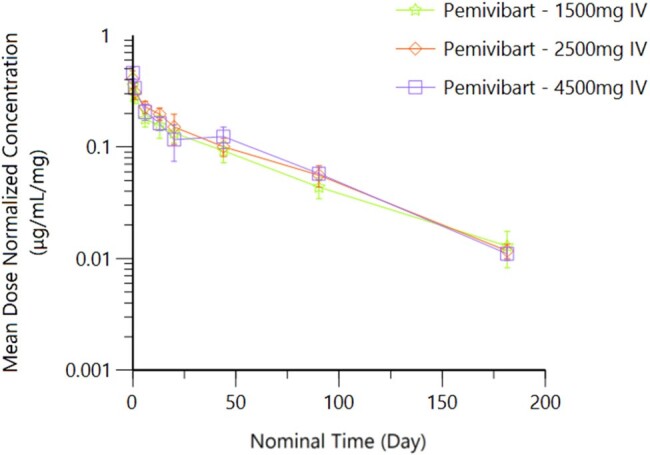
Mean ± Standard Deviation Serum Pemivibart Dose-Normalized Concentration-Time Profile

**Results:**

Thirty ppts were randomized (24 PEM, 6 PBO); 28 ppts received the full dose as planned; two ppts received approximately 93% of the full dose of study drug due to an administration error. In the PEM arm, the median age was 32.5 years, 13.3% of ppts were 55 years or older, most ppts were white (83.3%) and not Hispanic or Latinx (93.3%). Mean body mass index was 25.78 kg/m^2^ across all ppts and similar between cohorts and study arms. There were no deaths, serious adverse events (SAEs), or AEs leading to permanent study drug discontinuation. Infusion-related adverse events occurred in 4 ppts (2 each in Cohort 2 and 3, respectively); these events were self-limited and resolved within 5 minutes without treatment. No unexpected safety signals were observed. PEM demonstrated linear PK with apparent dose-proportional exposure and extended serum half-life (mean 46, 44, and 50 days in Cohort 1, 2, and 3, respectively [Table 1]). No substantial anti-drug antibodies (ADAs) have been observed.

**Conclusion:**

In this Phase 1 study of PEM administered by slow IV push at doses up to 4500 mg, no AEs leading to study drug discontinuation, or SAEs were reported to date in healthy adults. PEM demonstrated linear and dose-proportional PK. Complete trial data to be presented.

**Disclosures:**

Anna Holmes, PhD, Invivyd, Inc.: employee|Invivyd, Inc.: Stocks/Bonds (Private Company) Yong Li, PhD, Invivyd, Inc.: Employee|Invivyd, Inc.: Stocks/Bonds (Public Company) Deepali Gupta, BSc, Invivyd, Inc.: Employee|Invivyd, Inc.: Stocks/Bonds (Public Company) Ed Campanaro, MS, Invivyd, Inc.: Employee|Invivyd, Inc.: Stocks/Bonds (Public Company) Lida Mehr, B.S., Invivyd, Inc.: Advisor/Consultant Anuja Raut, M.S., Invivyd, Inc.: Advisor/Consultant Amanda Copans, Pharm.D., Invivyd, Inc.: Employee|Invivyd, Inc.: Stocks/Bonds (Public Company) Kristin Narayan, Ph.D., Invivyd, Inc.: Employee|Invivyd, Inc.: Stocks/Bonds (Public Company) Kathryn Mahoney, PharmD, Invivyd, Inc.: Employee|Invivyd, Inc.: Stocks/Bonds (Public Company)

